# Protein Quality Control of NKCC2 in Bartter Syndrome and Blood Pressure Regulation

**DOI:** 10.3390/cells13100818

**Published:** 2024-05-10

**Authors:** Kamel Laghmani

**Affiliations:** 1 Centre de Recherche des Cordeliers, INSERM, Sorbonne Université, Université de Paris, F-75006 Paris, France; kamel.laghmani@crc.jussieu.fr; 2 CNRS, ERL8228, F-75006 Paris, France

**Keywords:** kidney, NKCC2, protein quality control, ERAD, MAGE-D2, trafficking, Bartter syndrome, CKD, hypertension

## Abstract

Mutations in NKCC2 generate antenatal Bartter syndrome type 1 (type 1 BS), a life-threatening salt-losing nephropathy characterized by arterial hypotension, as well as electrolyte abnormalities. In contrast to the genetic inactivation of NKCC2, inappropriate increased NKCC2 activity has been associated with salt-sensitive hypertension. Given the importance of NKCC2 in salt-sensitive hypertension and the pathophysiology of prenatal BS, studying the molecular regulation of this Na-K-2Cl cotransporter has attracted great interest. Therefore, several studies have addressed various aspects of NKCC2 regulation, such as phosphorylation and post-Golgi trafficking. However, the regulation of this cotransporter at the pre-Golgi level remained unknown for years. Similar to several transmembrane proteins, export from the ER appears to be the rate-limiting step in the cotransporter’s maturation and trafficking to the plasma membrane. The most compelling evidence comes from patients with type 5 BS, the most severe form of prenatal BS, in whom NKCC2 is not detectable in the apical membrane of thick ascending limb (TAL) cells due to ER retention and ER-associated degradation (ERAD) mechanisms. In addition, type 1 BS is one of the diseases linked to ERAD pathways. In recent years, several molecular determinants of NKCC2 export from the ER and protein quality control have been identified. The aim of this review is therefore to summarize recent data regarding the protein quality control of NKCC2 and to discuss their potential implications in BS and blood pressure regulation.

## 1. Introduction

NKCC2 is a member of the superfamily of cation-coupled chloride cotransporters (CCCs), which is composed of three Na^+^-dependent chloride cotransporters (NKCC1, NKCC2, and NCC) and at least four Na^+^-independent K^+^-Cl^−^ cotransporters (KCC1-4) [1]. All members of this SLC12A family share the same hydropathy profile, with twelve transmembrane domains and two cytoplasmic N-terminal and C-terminal tails [1]. In contrast, to the widely expressed basolateral Na-K-2Cl isoform NKCC1 [1,2], NKCC2 is specifically expressed at the apical side of kidney TAL cells. This kidney-specific Na-K-2Cl cotransporter is considered the master player of NaCl reabsorption in the TAL, a key nephron segment responsible for the reabsorption of nearly 30% of filtered NaCl [3,4]. Sodium and chloride entering the TAL cells through NKCC2 exit these cells at the basolateral membrane via the Na^+^/K^+^-ATPase and Cl^–^ channels (CLC-KB), respectively [4,5]. In contrast to Na and Cl reabsorption, potassium carried into the cell through NKCC2 is recycled into the lumen via ROMK to preserve the correct functioning of the cotransporter [4]. Notably, this luminal K recycling generates a positive transepithelial voltage that promotes the absorption of Na^+^, Ca^2+^, and Mg^2+^ through the paracellular pathway [4]. Moreover, given that the TAL is impermeable to water, Na-Cl absorption in this segment of the nephron mediated by NKCC2 represents the central element in the generation of the osmotic gradient that is required for the urinary concentrating mechanism [6]. As a result, even subtle modifications in cotransporter transport activity, directly related to the protein itself or its regulators, may have a major impact on several kidney functions. Indeed, mutations in NKCC2 lead to type 1 BS, a serious renal disorder usually diagnosed prenatally by the detection of polyhydramnios due to fetal polyuria, salt wasting, and electrolyte abnormalities [1,7]. In contrast to the genetic loss of function of NKCC2, an inappropriate increase in NKCC2 transport activity has been linked to salt-sensitive hypertension [8,9]. Accordingly, furosemide and bumetanide, two pharmacologic inhibitors of NKCC2, are widely used as loop diuretics to lower blood pressure (BP) in hypertensive patients [10]. All of these data highlight the fundamental role of NKCC2 in electrolyte homeostasis and BP control in normal physiology and the pathogenesis of BS. In recent years, efforts to better characterize NKCC2 function and regulation have revealed that it is essential to elucidate the underlying mechanisms and respective protein networks governing NKCC2 expression at the molecular level to elucidate the pathophysiology of type 1 BS and explore new options to improve the available treatments for this salt-losing tubulopathy and salt-sensitive hypertension. Virtually all previous reviews on the molecular regulation of this cotransporter have focused primarily on the post-Golgi regulation of NKCC2, notably by subcellular redistribution (endocytosis and exocytosis) and phosphorylation [11,12,13,14,15,16]. The present article is the first review that focuses on the protein quality control of NKCC2, particularly the ER quality control (QC) of the cotransporter, and discusses its relevance to salt-losing tubulopathy and blood pressure regulation.

## 2. NKCC2 Expression in Mammalian Cells: It Is All about Protein Quality Control

The cDNA encoding NKCC2 was identified in 1994 by two different groups [17,18]. Additionally, it was revealed that there are three splice variants of NKCC2, named NKCC2-A, NKCC2-B, and NKCC2-F [18]. NKCC2F is expressed exclusively in the medullary TAL [19,20,21]. NKCC2A was found in both medullary and cortical TAL and macula densa (MD) cells [19,20,21]. NKCC2B is expressed primarily in MD cells and the cortical portion [19,20,21]. Of note, considering the pivotal role of NKCC2 in NaCl reabsorption in the TAL, especially in the countercurrent multiplier system, NKCC2-F seems to be more important than NKCC2-A and NKCC2-B [20,22]. In support of this idea, NKCC2 knock-out mice die shortly after birth due to severe polyuria [23], whereas NKCC2B and NKCC2A null mice were viable and exhibited only very wild phenotypes [24,25].

Despite the cloning and identification of NKCC2 cDNA since the mid-1990s, efforts to achieve the functional expression of NKCC2 protein in cultured mammalian cells have failed for over a decade, and regulatory characterizations of NKCC2 proteins have been limited to Xenopus laevis oocytes [26]. Therefore, our knowledge of the molecular regulation of protein QC and the membrane trafficking of wild-type (WT) NKCC2 and its mutants in mammalian cells, particularly through protein-protein interactions, has remained very poor for years. Interestingly, unlike full-length NKCC2, immunoreactive proteins could be detected by Western blotting in cells transiently transfected with NKCC2 deprived of the first 105 AAs of its N-terminus [18]. Additionally, it was also possible to express an NKCC1-NKCC2 chimera in which the first 104 amino acids of NKCC2 were substituted with the equivalent region from NKCC1 [27,28]. Based on these observations, we postulated that the proximal region of the NKCC2 N-terminal tail plays a crucial role in protein QC and thus in the stability of the cotransporter protein. Consistent with this notion, several studies reported that tagging the N-terminus of certain proteins blocked their degradation, very likely by preventing the N-terminal ubiquitination pathway [29,30,31]. Hence, we anticipated that it would be possible to express NKCC2 protein by tagging its N-terminal domain, notably with Myc or GFP. Using this strategy, my group was the first to be able to stably express NKCC2 protein (NKCC2-F) in several cultured mammalian cells, such as MKTAL cells, OKP cells, and HEK cells [9,32,33,34], providing us, therefore, with a powerful tool to study and understand the molecular mechanisms underlying the biogenesis and regulation of the cotransporter in kidney cells. This powerful heterologous expression system allowed us to particularly study the protein QC of the cotransporter, especially during its transit through the endoplasmic reticulum (ER).

## 3. Molecular Determinants of NKCC2 ER Export

To enable the TAL to make appropriate adaptations to physiological or pathological challenges, NKCC2 must function properly and therefore must be correctly and sufficiently trafficked to the plasma membrane. Since it is a transmembrane protein, the appropriate forward trafficking of NKCC2 to the cell surface begins when the cotransporter protein is inserted in the ER, and then continues through the Golgi and post-Golgi networks. The tightly controlled delivery of membrane proteins to the cell surface is dictated by specific targeting motifs within the protein. Multiple ER exit motifs, such as adjacent hydrophobic residues (FF, FY, LL, IL, or YYM) and diacid codes, or a combination of several sorting signals, have been identified within the cytoplasmic carboxy terminus of numerous transmembrane proteins [35,36]. Moreover, several genetic disorders are caused by COOH-terminal mutations [37,38]. Consistent with this, type 1 BS mutations D918fs, N984fs, and Y998X, within the carboxy-terminal tail of NKCC2, are not functional because they are not delivered at the plasma membrane because they are retained in the ER [33]. Hence, we took advantage of the existence of naturally occurring mutants, deleting the distal region of the NKCC2 COOH-terminal tail to determine the ER key exit motif(s) of the cotransporter.

### 3.1. Di-Leucine-like Motifs

**Figure 1:**
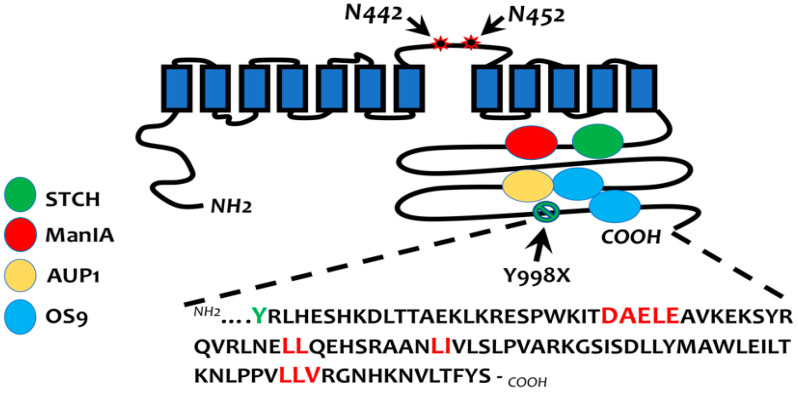
Sorting signals and binding partners of NKCC2 at the ER. To identify NKCC2 binding partners involved in the protein QC of the cotransporter, we previously used the Y2H system for the screening of a human cDNA library using three regions of the NKCC2 COOH terminus as bait [32]. While STCH and ManIA bind to the proximal region of the NKCC2 C-terminal tail [42,43], AUP1 interacts with the intermediate region [44]. OS9 binds to both the intermediate [44] and distal regions of the NKCC2 carboxy-terminal tail [45]. To identify the ER export signals of NKCC2, we focused on the region downstream from the Y998X mutant that harbors several di-leucine-like motifs and consensus di-acidic (D/E-X-D/E) codes. The motifs required for ER export from the ER are in red.

To identify the ER exit signals(s) of NKCC2, we focused on the region downstream from Y998X, the most distal natural mutation identified in the carboxy-terminal tail of NKCC2 (Figure 1), as the key sequence governing the export of NKCC2 from the ER. Using progressive truncations and mutagenesis studies in this region, we demonstrated that ^1038^LL^1039^,^1048^LI^1049^, and ^1081^LLV^1083^ hydrophobic motifs are required for NKCC2 exit from the ER (Figure 1), independently of the expression system (OKP and HEK cells) [33,39]. Mutating these motifs to alanine residues prevented ER exit and the subsequent surface expression of NKCC2 [33,39]. Importantly, among all analyzed di-leucine-like motifs that are present downstream from Y998X (Figure 1), only those required for NKCC2 ER exit are remarkably highly conserved among SLC12A family members, strongly suggesting that they may function as common ER export signals for all CCCs. In support of this notion, mutating the trihydrophobic motif (LLI) within the C-terminal of NCC, the corresponding ^1081^LLV^1083^ motif of NKCC2, also prevented the ER exit of this structurally related renal Na-Cl cotransporter [33]. These results are particularly interesting given that CCCs are involved in several pathologies such as Bartter, Gitelman, and Anderman syndromes [1,40]. These results therefore open the possibility that the loss of these highly conserved evolutionary hydrophobic motifs could contribute to the development of several pathologies linked not only to kidney function but also to processes in which other members of the CCC family are involved, such as neuronal functions [40,41].

### 3.2. Di-Acidic Codes

Although the highly conserved hydrophobic motifs described above are required for NKCC2 exit from the ER, and thus may serve as ER export signals, one cannot rule out the possibility that they may also provide sites for interaction of the cotransporter with the cellular machinery that assists the protein in reaching a folding state competent for transport out of the ER. In line with this alternative, mutating the ILLV motif within the NKCC1-COOH-terminus [46], which contains the corresponding ^1081^LLV^1083^ motif of NKCC2, causes the misfolding and protein aggregation of this basolateral Na-K-2Cl cotransporter. Consequently, this prompted us to further closely analyze the distal region of the NKCC2 C-terminal tail to check for the presence of a pure ER export signal. Interestingly, this sequence analysis unveiled the presence of consensus di-acidic (D/E-X-D/E) motifs, ^1019^DAELE^1023^, located downstream from Y998X (Figure 1). In contrast to di-leucine-like motifs, di-acidic codes are generally considered pure ER export signals. Importantly, the triple mutation of the D, E, and E residues of ^1019^DAELE^1023^ to ^1019^AAALA^1023^ prevented the ER exit of NKCC2, therefore abolishing its complex glycosylation and cell surface expression [47]. Given that di-acidic codes are implicated in the ER export of cargo proteins via the COPII budding machinery, the effect of overexpressing Sar1^H79G^, a dominant negative form of the small Sar1 GTPase, was studied. Sar GTPase is the initiator of COPII-coated vesicle formation. Importantly, in cells overexpressing Sar1^H79G^, NKCC2 proteins failed to reach the plasma membrane because they were trapped in the ER, clearly demonstrating the requirement of COP-II machinery for the ER export of the cotransporter [47]. Collectively, these results revealed that the ER export of NKCC2 is COP-II-dependent and is controlled by a combination of several sorting signals, including highly conserved hydrophobic motifs and di-acidic exit codes. It should be noted that these different sorting motifs may operate coordinately or independently to ensure the proper delivery of NKCC2 to the cell surface. Based on these observations, we proposed that any mutation in NKCC2 that interferes with these sorting signals and/or the COP-II interaction with the cotransporter could constitute the molecular basis of type 1 BS under such circumstances.

## 4. ERAD Components of NKCC2

ER QC is the process that tightly controls newly synthesized membrane and secretory proteins in the ER and prevents them from being transferred to the Golgi network before being properly folded [48,49,50]. The ER accomplishes this function through molecular chaperones, which are capable of recognizing protein-folding defects [51]. Permanently misfolded proteins are trapped in the ER before being targeted for ER-associated protein degradation (EARD) in the cytoplasm [48,49,50]. The ERAD machinery is therefore devoted to the clearance of unwanted misfolded proteins, as the accumulation of misfolded protein aggregates in the ER can have detrimental effects on cellular health [49]. Similar to several other membrane proteins with complex topologies, such ENaC [52] and CFTR [53], the vast majority of newly synthesized (around 70%) NKCC2 proteins are targeted for ERAD [39]. NKCC2 maturation is a relatively slow and inefficient process, strongly suggesting that export from the ER constitutes the rate-limiting step in the NKCC2 journey to the cell surface. Accordingly, identifying NKCC2 binding partners in the ER is crucial to elucidate the molecular pathway by which the cotransporter is subject to QC. To uncover NKCC2 interactors, we opted for a yeast two-hybrid system (Y2H) approach and used a series of baits covering the predicted NKCC2 COOH terminus to screen a human kidney cDNA expression library [32]. This strategy allowed us to unveil the identity of several components of NKCC2 quality control. 

### 4.1. Interaction with OS9 and AUP1

Using Y2H, the ER-lectin OS9 (Osteosarcoma amplified 9) and AUP1 (Ancient Ubiquitous Protein 1) were identified as novel interactors in the NKCC2 COOH terminus (Figure 1) [44,45]. This is of great importance because interaction between AUP1 and OS9 has been reported [54], opening, therefore, the possibility for a tripartite complex between these two proteins and NKCC2. The main function of OS9 remained unclear until human OS9 was reported to be implicated in ER-to-Golgi transport and the ERAD of misfolded glycoproteins [55,56,57,58]. Similarly, while initial reports described AUP1 as a cytosolic protein with a role in integrin signaling [52], more recent studies have revealed that AUP1 is localized to the ER membrane and plays a crucial role in the ERAD system, in particular by promoting the polyubiquitination of proteins [47,53,54]. Given AUP1’s hairpin topology in the ER membrane, with the N and C tails of the protein facing the cytosol [59], and that OS9 might be present at both the luminal and cytoplasmic sides of the ER [55,60], an interaction between these proteins and NKCC2 at the cytoplasmic surface of the ER is conceivable. Using two different kidney cell lines (HEK and OKP cells), we provided evidence that both OS9 and AUP1 interact with the ER-resident form of NKCC2, which is coherent with a potential role in the ERAD of the cotransporter [44,45]. Accordingly, OS9 and AUP1 overexpression decreased the stability and the maturation of NKCC2, whereas their knockdown produced the opposite effect. Mutating NKCC2 *N*-glycosylation sites or inhibiting mannose-trimming with kifunensine completely abolished the effects of OS9 and AUP1 on NKCC2, indicating that the enhanced degradation of the cotransporter is *N*-glycan-dependent. Importantly, AUP1 and OS9 influences on immature NKCC2 were also abrogated by MG132, suggesting that OS9 and AUP1 target the core glycosylated form of NKCC2 to the proteasome-dependent ERAD pathway [44,45]. Furthermore, in the presence of MG132, the increase in NKCC2 protein amount upon the inhibition of endogenous AUP1 was associated with a striking decrease in polyubiquitinated NKCC2 species, demonstrating that AUP1 enhances cotransporter ERAD by promoting its polyubiquitination [44]. Notably, although the effects of AUP1 and OS9 on NKCC2 were similar, they were not additive, strongly suggesting that both proteins work on the same molecular pathway to regulate the ERAD of NKCC2. However, one cannot predict at this stage whether they interact sequentially or simultaneously with the cotransporter. Indeed, one alternative possibility is that, similar to Hsp40 and Hsp70 [51], we may also have a substrate (here, NKCC2) transfer from OS9 to AUP1, or vice versa. Regardless of the nature of AUP1’s and OS9’s cooperation to control NKCC2 expression, it is tempting to speculate that NKCC2 regulation by these two binding partners may also take place in native TAL cells and thereby contribute to the chronic adaptations of the cotransporter. In support of such a possible mechanism of NKCC2 regulation, patients with an abnormal increase in the level of uromodulin, a protein partner of NKCC2, developed furosemide-sensitive salt-dependent hypertension due to an inappropriate increase in the cotransporter’s protein expression [9]. Analogously, modulations of AUP1 or OS9 protein levels could also alter NKCC2 abundance and function in vivo. In this regard, both proteins are upregulated during ER stress and promote the ERAD of glycoproteins under these conditions [58,61]. Overexpression studies of AUP1 and OS9 may therefore mimic their upregulation under circumstances of ER stress in kidney cells, for example, in chronic high dietary salt intake and aging, during which NKCC2 is downregulated [62,63,64,65]. Accordingly, the upregulation of AUP1 and/or OS9 might participate in cotransporter downregulation through the ubiquitin–proteasome system (UPS), especially under ER stress circumstances [64,65]. In this regard, it should be noted that the role of the UPS in the regulation of BP, in particular by regulating sodium transporters in the kidney, is now well documented [66].

### 4.2. Interaction with STCH and Hsp70

**Figure 2:**
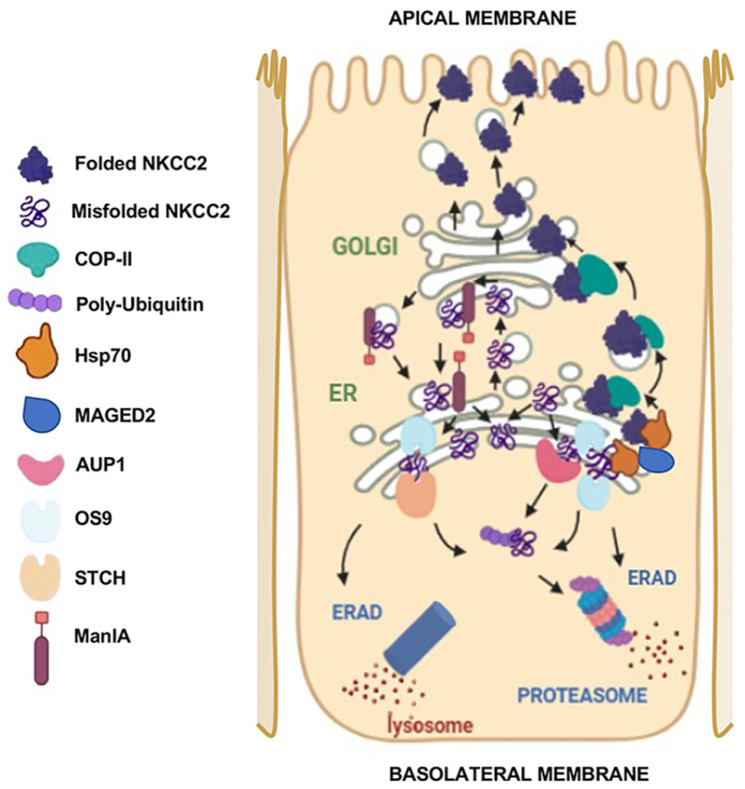
Molecular determinants of NKCC2 ER exit and protein quality control of NKCC2. ER export from the ER of correctly folded NKCC2 proteins is COPII COPII-dependent and requires a combination of multiple sorting signals such as di-acidic codes and di-leucine-like motifs. OS9 promotes the ER retention of misfolded NKCC2 proteins and their degradation by the proteasome pathway. AUP1 enhances the proteasome-dependent ERAD of NKCC2 by promoting its polyubiquitination. Besides the proteasome pathway, STCH promotes NKCC2 ERAD via the lysosome pathway. Through Golgi ManIA, Golgi QC serves as an additional checkpoint to remove misfolded NKCC2 proteins that escaped ER QC surveillance by capturing them at the Cis-Golgi network and delivering them back to the ER. Hsp70 protects NKCC2 against ERAD and promotes its maturation. Finally, MAGE-D2 promotes NKCC2 biogenesis, most likely through its interaction with Hsp70 and/or its cochaperone Hsp40.

In addition to AUP1 and OS9, the Y2H screening identified the stress 70 protein chaperone (STCH), a constitutively expressed member of the Heat shock protein 70 (Hsp70) family, as a novel NKCC2-interacting protein [42]. Similar to AUP1 and OS9, the idea of the possible involvement of STCH in the ERAD of NKCC2 emerged from the fact that the interaction recruited only the immature and ER-resident forms of NKCC2. STCH overexpression and knock-down studies in OKP and HEK cells revealed that STCH indeed enhances the ERAD of the cotransporter. Unexpectedly, unlike AUP1 and OS9, the STCH effect on NKCC2 was not completely abolished upon proteasome inhibition by MG132 [42]. The complete recovery of NKCC2 protein levels after STCH overexpression was only achieved by the simultaneous presence of MG132 and chloroquine, a lysosomal inhibitor, thus revealing the requirement of the lysosome for the elimination of misfolded NKCC2 in these situations [42]. The necessity of additional and complementary ER QC pathways, defined as ER-associated lysosome degradation (ERLAD) pathways, gradually emerged, as some misfolded proteins escape the ERAD system [67,68]. Also, some misfolded ER clients form aggregates that are not suitable for classical ERAD, and therefore their removal from the ER relies on the lysosomal alternative via autophagic or non-autophagic pathways. In this regard, Hsp70 chaperones are thought to have a dual role in protein degradation by being involved in the clearance of misfolded proteins via the proteasome and lysosome pathways [69,70]. Fittingly, STCH, a constituent of the Hsp70 system, is here implicated in the efficient removal of unwanted NKCC2 proteins via these two complementary pathways (Figure 2). Hence, in addition to the ERAD machinery, the characterization of ERLAD as an alternative pathway for NKCC2 degradation opens new avenues in studying the molecular mechanisms underlying the protein QC of NKCC2.

These findings on STCH are the first demonstrating its direct involvement in the ERAD of misfolded proteins [42]. Given that the stress-inducible member of the Hsp70 family Hsp-70-1, commonly known as Hsp70, is also implicated in the ERAD system [69,71,72], the STCH effect was compared with that of Hsp70 on NKCC2 biogenesis. Similar to STCH, Hsp70 binds only to the ER-resident form of NKCC2. However, STCH and Hsp70 were found to exert opposite effects on the cotransporter. Indeed, while STCH enhances NKCC2 ER retention and degradation, Hsp70 promotes the maturation of the cotransporter (Figure 2). Regarding this, it is important to mention that in the context of cellular stress, Hsp70 preferentially promotes the correct folding and stabilization of newly synthesized proteins [73]. In contrast, it exerts the opposite effects under non-stress conditions [73]. This is of great interest because the exogenous overexpression of membrane proteins (such as NKCC2) generates ER stress, which is thereby coherent with the Hsp70 protective role during the ERAD of the cotransporter in these circumstances. Of note, while the relevance of STCH and Hsp70 interaction with NKCC2 in TAL physiology remains to be determined, it remains plausible to anticipate that changes in their expression in vivo may have an impact on the cotransporter’s abundance and function. For instance, several studies have reported that the chronic infusion of angiotensin II, a model of salt-sensitive hypertension, induces the expression of Hsp70 in renal cells, which can contribute thereby to the upregulation of NKCC2 observed under the same conditions [74,75,76].

### 4.3. Interaction with Golgi ManIA

Intriguingly, the Y2H screening followed by a co-immunoprecipitation assay revealed that Golgi alpha1,2-mannosidase IA (ManIA) is also an interactor of the high-mannose glycosylated form of NKCC2 [43]. ManIA is a type II transmembrane protein with a cytoplasmic N-terminus tail and a luminal COOH-terminus [77]. Since ManIA was identified as an interactor of the C-terminal tail of NKCC2, its association with the cotransporter likely involves its cytoplasmic N-terminus tail. Moreover, given that the interaction of the proteins engages only the core glycosylated form of NKCC2 and that ManIA is localized either in the Golgi and/or in the ER [78,79,80], one can postulate that the primary site of the interaction is the ER. Nevertheless, since the N-glycan of glycoproteins at the entrance to the Golgi complex is still of high-mannose-type, an interaction of NKCC2 with ManIA in the cis-Golgi network is also conceivable. Importantly, co-localization experiments revealed that the principal site of interaction of NKCC2 with ManIA is the cis-Golgi network, regardless of the heterologous protein expression system used (OKP and HEK cells) [43]. Remarkably, overexpression and siRNA studies unveiled that, similar to AUP1 and OS9, ManIA targets immature NKCC2 to the proteasome-dependent ERAD pathway [43]. Notably, deleting the cytoplasmic tail of ManIA, which is presumably the essential domain of interaction with NKCC2, inhibited ManIA binding and abrogated its impact on the cotransporter’s maturation. Furthermore, deleting the catalytic domain of ManIA completely prevented its influence on the fate of NKCC2 [43]. Collectively, these data accumulate evidence that Golgi ManIA promotes efficient ER retention and the associated degradation of misfolded NKCC2 proteins. They are in line with the notion that the Golgi apparatus serves also as an important protein QC checkpoint [81]. Hence, the results described above are consistent with a model whereby, through Golgi-ManIA, cis-Golgi QC serves as an additional checkpoint for the protein QC of NKCC2 by promoting the retention, recycling, and proteasome-dependent ERAD of misfolded NKCC2 proteins that initially escape surveillance within the ER (Figure 2).

## 5. Plasma Membrane Quality Control of NKCC2

To preserve protein homeostasis, eukaryote cells rely on multiple and successive QC mechanisms, including ER QC, Golgi QC, and plasma membrane QC [81]. Under certain circumstances, particularly under conditions of ER stress, these different QC pathways must cooperate to efficiently remove unwanted and harmful proteins. NKCC2 is no exception, and therefore, besides ER and Golgi QC, NKCC2 is also subject to a quality control process at the plasma membrane. In an elegant study using native TAL cells, Ares, G. provided evidence that surface NKCC2 protein is constitutively ubiquitinated and regulated by the proteasome pathway, a process that is stimulated by cGMP [82]. This is of particular interest because cGMP is an important regulator of NaCl in the TAL by decreasing NKCC2 cell surface expression [83]. Interestingly, cGMP-induced NKCC2 ubiquitination and degradation require a cullin-RING ligase (CRL) complex. Inhibiting CRL complex regulators, such as Nedd8 and CAND1, affects cGMP-dependent NKCC2 ubiquitination, and thereby the cell surface expression of the cotransporter. In support of the UPS-mediated regulation of NKCC2, and thereby in relation to BP control, Wu et al. provided also evidence for the role of UPS in the regulation of NKCC2 abundance during high salt intake [64]. Indeed, using CYP4F2 transgenic mice, they reported that 20-HETE and high salt intake synergistically diminish NKCC2 protein expression via the Nedd4-2-mediated ubiquitin-proteasome pathway, and thereby regulate natriuresis and BP [64].

## 6. Molecular Basis of Bartter Syndrome Type 1

As mentioned above, type 1 BS is a serious kidney disorder caused by inactivating mutations in NKCC2. The presenting features of this inherited tubulopathy include polyhydramnios secondary to polyuria, polydipsia, growth retardation, massive salt wasting generating hypotension, hypokalemic metabolic alkalosis, hypercalciuria, and nephrocalcinosis [84,85]. To date, BS is a treatable disease, but still incurable. Furthermore, in the long term, the prognosis of BS is dominated by the possibility of chronic renal failure. Therefore, in-depth investigations into the molecular mechanisms underlying NKCC2 expression in renal cells are paramount to elucidate the pathophysiology of BS1 and to upgrade treatments for the disease. Incontestably, only the analysis of NKCC2 mutants in renal cells would make it possible to clearly and definitively determine their cellular fate.

### 6.1. Molecular Pathogenic Mechanisms of NKCC2 Mutations

To date, more than 63 mutations in NKCC2 have been described in BS individuals, with highly variable phenotypic expression and clinical severity [84]. Despite this, only seven type 1 BS1 mutations have so far been studied in mammalian cells. Three of these mutations are the truncating mutations (D918fs, N984fs, and Y998X) discussed above. In this section, I will focus on the molecular pathogenic mechanisms of NKCC2 missense mutations.

Using our heterologous expression system (OKP and HEK cells), we recently examined the effect of four NKCC2 missense mutations (E368G, Y477N, A508T, and A628D) on NKCC2 transport function and processing [86]. We observed an altered transport activity with these NKCC2 variants, thus explaining the defect in TAL NaCl reabsorption in patients carrying these mutations [86]. In general, there are several mechanisms by which mutations could impair NKCC2 transport activity, such as impaired protein synthesis, disturbed protein processing, enhanced protein degradation, defective insertion into the plasma membrane, and an alteration of the functional properties of the cotransporter. Our data unveiled that all NKCC2 variants are not expressed at the cell surface because they are retained in the ER. Moreover, treatment with proteasome and lysosome inhibitors failed to restore the loss of the complex-glycosylated and mature forms of NKCC2, thus further ruling out the possibility that the Golgi apparatus processes NKCC2 mutants [86]. As mentioned above, we proposed abnormal NKCC2 maturation as a common type 1 BS mechanism associated with mutations such as D918fs, N984fs, and Y998X, depriving NKCC2 of its COOH terminus. The results obtained with NKCC2 missense mutations further supported the notion that, although other molecular mechanisms may contribute to the development of type 1 BS, ER retention and ER-associated protein degradation seem to be the major mechanisms underlying the disease. Importantly, similar to WT NKCC2, the ERAD of NKCC2 mutants is also mediated by the ER lectin OS9, AUP1, STCH, and ManIA [42,43,44,86]. More importantly, these specific constituents of NKCC2 ERAD machinery had a more profound effect on the cotransporter’s folding mutants when compared to WT NKCC2. These results strongly suggest, therefore, that during the disease state, OS9, AUP1, STCH, and ManIA work in concert, sequentially, or simultaneously to efficiently eliminate harmful misfolded NKCC2 proteins and aggregates, thereby protecting TAL cells from proteotoxicity.

### 6.2. Rescuing NKCC2 Mutants: Potential Strategies

The results described above demonstrate that type 1 BS is one of the diseases associated with the ERAD pathway. The absence of functional activity and cell surface expression of these mutants is very likely secondary to folding defects and, thereby, to ER retention and degradation mechanisms. Given that all the studied mutations did not concern the active site of the cotransporter, a therapeutic strategy allowing the correction of the abnormal folding and trafficking of these NKCC2 mutants could allow the pharmacological treatment of the disease. Thus, we attempted to rectify the processing defect of NKCC2 mutants using chemical and molecular chaperones. The results were promising given that it was possible to rescue, at least partially, the maturation of NKCC2 folding mutants by chemical chaperones, in particular by glycerol, and to a lesser extent by the ER stress reliever 4-PBA [86]. Unfortunately, the observed effects on the mature and functional form of NKCC2 mutants were weak, and it was therefore not possible to completely rescue the expression of the cotransporter [86]. Similarly, OS9 knockdown massively increased the expression of NKCC2’s immature form but failed to rescue the mature form of the cotransporter [86]. In this regard, numerous studies on the CFTR ΔF5408 mutant, which is responsible for cystic fibrosis, have accumulated evidence that the strategy of combining multiple compounds is very successful in fully rescuing the processing of CFTR mutants [87,88,89]. Accordingly, more intensive investigations are needed to fully rescue NKCC2 mutants, very likely by testing combinations of several chemical chaperones and/or the simultaneous siRNA of several molecular chaperones, such as Golgi ManIA, STCH, OS9, and AUP1.

## 7. Molecular Mechanisms of Bartter Syndrome Type 5

Phenotypically, BS is divided into two subgroups: antenatal BS (aBS) and classical BS. aBS is considered the most severe form of the disease, characterized by polyhydramnios and prematurity due to severe fetal polyuria [85]. Genotypically, in addition to type 1 BS, four other types of the disease due to mutations in ROMK (type 2), ClCKB (type 3), Barttin (type 4), and MAGE-D2 (type 5) have been described [85]. Type 5 BS is the most severe form of the disease, as illustrated by its high mortality due to a very early onset of severe polyhydramnios and extreme prematurity [90]. Type 5 BS is also called transient aBS because patients with this disease show a complete resolution of symptoms at the end of the pregnancy or just after birth [90]. In this section, I will focus on type 5 BS, the most recent and severe form of aBS, and the implications of NKCC2 in the disease.

### 7.1. MAGE-D2: A Key Modulator of NKCC2 Biogenesis

Despite the identification of several forms of BS, at least one-fifth of patients with aBS and hypercalciuria, the signature symptom of NKCC2 dysfunction, remained without a genetic explanation for decades [85,91]. This led to the hypothesis of the involvement of indirect mechanisms underlying the disease, for example, the mutation of a regulatory protein of NKCC2 that could alter its trafficking toward the plasma membrane and its transport activity, thus inducing in the patient a phenotype similar to type 1 BS. Additionally, a transient form of aBS has been described, but its molecular basis remained unknown for years.

In the context of identifying novel molecular pathways responsible for aBS, whole-exome sequencing analysis was performed in a family in which three pregnancies with male offspring were complicated by transient aBS. This led to the identification of mutations in the MAGE-D2 (melanoma-associated antigen D2) gene, which correlated with the disease [90]. MAGE-D2 encodes melanoma-associated antigen D2 and maps to the X chromosome. In total, nine different mutations in MAGE-D2 were identified in the initial report [90]. Following this initial study, Legrand et al. reported that MAGE-D2 mutations were the cause of at least 9% of cases in a French cohort containing 171 families of patients with aBS [92]. Since then, the MAGE-D2 gene has been systematically included in the genetic screening of severe clinical forms of aBS [93,94].

The phenotypic overlap between type 1 BS and type 5 BS suggested a defect in NKCC2 expression in patients carrying MAGE-D2 mutations. In support of this, immunohistochemistry analysis revealed that NKCC2 was not detectable at the TAL apical membrane of a stillborn fetus carrying a MAGE-D2 mutation due to the ER retention of the cotransporter. These findings again support the notion that the ER export from the ER constitutes the limiting and crucial step in the forward trafficking of NKCC2 to the apical membrane. However, a defect in NKCC2 expression cannot alone explain the severity of the disease. This led to the analysis of NCC expression, which is the key actor in NaCl reabsorption in the distal tubule. NKCC2 and NCC belong to the same family of CCCs, and are often regulated by the same molecular mechanisms [1]. Importantly, NCC was also not detected at the apical membrane of the distal tubule in patients carrying MAGE-D2 mutations, due to ER retention. Simultaneous defects in NKCC2 and NCC expression could therefore explain, at least in part, the severity of aBS in patients with MAGE-D2 mutations.

### 7.2. Molecular Basis of the Transient Nature of BS Type 5

To elucidate the mechanisms underlying NKCC2 regulation by MAGE-D2, in vitro studies were conducted in HEK cells. These experiments revealed that MAGE-D2 knock-down reduces the stability and maturation of the cotransporter, while its overexpression produces the opposite effect [90]. Furthermore, the overexpression of MAGE-D2 increased the membrane expression and transport activity of NKCC2. Interestingly, the magnitude of the increase in the NKCC2 cell surface level by MAGE-D2 exceeded that observed on the stability and maturation of the cotransporter, thus opening the possibility for the implication of MAGE-D2 in the regulation of NKCC2 post-Golgi trafficking. Collectively, these data strongly suggest therefore that (1) MAGE-D2 plays a protective role against the ERAD of NKCC2 and (2) MAGE-D2 may regulate NKCC2 post-Golgi trafficking.

To further explore the molecular pathways involved in MAGE-D2’s effects on NKCC2 and NCC, the MAGE-D2 interactome was analyzed. This proteomic approach revealed that MAGE-D2 is a specific interactor of Hsp40 [90]. Interestingly, a more recent report also unveiled the interaction of MAGE-D2 with Hsp70 [95], which is an Hsp40 cochaperone and a protector of NKCC2 against ERAD [42]. Consequently, Hsp70 and Hsp40 are very likely to cooperate with MAGE-D2 to protect NKCC2 against the ERAD system. In addition to Hsp70 and Hsp40, MAGE-D2 also interacts specifically with GNAS (Gsα) [90]. This interaction is also very important because total cellular NKCC2 protein abundance was decreased in GNAS knockout mice [96], strongly suggesting that Gsα is also a key regulator of NKCC2 protein stability. Moreover, since cAMP is an enhancer of cell surface expression and the transport activity of NKCC2 and NCC [15,97], Gsα may also be implicated in the regulation of NKCC2 and NCC post-Golgi trafficking by MAGE-D2.

Regarding the transient character of the disease, one possibility is that, via its interaction with Hsp40/Hsp70 and/or Gsα, MAGE-D2 safeguards the cotransporters against cellular stress, which is very likely activated by the tissue hypoxia physiologically present in the fetal kidney during the first trimesters of pregnancy, and which resolves around 30 weeks of gestation alongside a spontaneous resolution of salt loss. The correction of tissue hypoxia at the end of pregnancy and/or during the postnatal period would thus contribute to the restoration of the expression of NKCC2 and NCC. At the same time, an increase in cAMP synthesis at the end of pregnancy and/or during the postnatal period could also contribute to the restoration of these two cotransporters’ expressions. Importantly, and in support of this possible scenario, Seaayfan et al. demonstrated recently that in hypoxic conditions, MAGE-D2 is required for the appropriate induction of HIFα, for the expression of Gsα at the cell surface, and thereby for activation of the cAMP pathway [98,99]. These results thus explain, at least in part, the transient nature of BS5, given that the salt transporters involved, NKCC2 and NCC, are activated by cAMP.

## 8. Phenotype Variability in Carriers of NKCC2 Mutations

Although the diagnosis of type 1 BS is usually made in the perinatal period, several reports have described patients in whom the disease was diagnosed only during puberty, demonstrating phenotypic variability in this renal tubular disorder [100,101,102]. Intriguingly, it was also reported that some heterozygous NKCC2 mutations in otherwise healthy individuals are, actually, protective against hypertension [103]. However, the functional characterization of those variants addressed by two independent groups that focused only on NKCC2-A revealed some discrepancies between their findings [104,105]. Consequently, the molecular mechanisms underlying NKCC2 regulation under these conditions are not fully understood. More importantly, the molecular basis of the phenotypic variability in carriers of NKCC2 mutations remains unknown. Given that the TAL principally expresses NKCC2-A and NKCC2-F, we raised the hypothesis that distinct NKCC2 mutations can differentially compromise the expression of these NKCC2 variants, providing therefore, at least in part, a rationale for the variability in symptoms seen in individuals who are carriers of NKCC2 mutations (29–33). Importantly, our results could support this idea by revealing, for instance, that NKCC2A-P348L exhibits only a partial loss of complex glycosylation, whereas NKCC2F-P348L exhibits only an immature form and is trapped in the ER [106]. Therefore, these data validate our hypothesis that physiological and pathophysiological challenges may differentially regulate the protein quality control of NKCC2 variants and, therefore, the processing of cotransporter mutants is governed by individual mutant-specific mechanisms. Elucidating how a specific challenge preferentially affects NKCC2 isoform A or F could provide new insights into the processes governing protein transport and their function relevant to kidney health and disease, in particular in Bartter syndrome and BP regulation.

## 9. Conclusions and Perspectives

The data and observations mentioned above are consistent with a model (Figure 2) whereby (1) di-acidic codes and hydrophobic di-leucine-like motifs dictate the ER exit of correctly folded NKCC2 via the COPII machinery; (2) the ER-lectin OS9 promotes the ER retention of the cotransporter and enhances its ER-associated degradation via the proteasome; (3) AUP1, an interactor of OS9, cooperates with the latter to promote the efficient ERAD of NKCC2 by enhancing its polyubiquitination; (4) STCH plays a dual function in the efficient clearance of misfolded NKCC2 proteins via the proteasomal and lysosomal pathways; (5) Golgi ManIA contributes to the efficient ERAD of the cotransporter by retaining in the cis-Golgi network misfolded NKCC2 proteins that have escaped ERQC, especially under ER stress conditions, and returning them to the ER; (6) in contrast to OS9, AUP1, ManIA, and STCH, Hsp70 protects NKCC2 against the ERAD system and improves its maturation; (7) Very likely through interaction with Hsp70 and its cochaperone Hsp40, MAGE-D2 promotes NKCC2 biogenesis. Further extensive studies are required to unveil the precise molecular mechanisms behind this model. Moreover, NKCC2 proteins at the plasma membrane are also modulated by the UPS system, a mode of regulation requiring a cullin-RING ligase complex [83]. In this regard, a better understanding of the molecular mechanisms underlying the plasma membrane QC of NKCC2, in particular by identifying the E3 ubiquitin ligases involved in the regulation of the cotransporter’s cell surface expression, could pave the way to the discovery of new and specific loop diuretics for the treatment of hypertension. Moreover, given that changes in NKCC2 expression occur with several chronic ER stress and hypertensive conditions, such as diabetes mellitus, obesity, and aging [65,107,108], changes in NKCC2-interacting proteins, in particular under ER stress conditions, could have an impact on the cotransporter’s abundance and function, and thereby on chronic kidney adaptations, especially the regulation of BP. Future research will benefit from the identification and characterization of additional regulators of NKCC2 and determine their physiological relevance in vivo by moving beyond studies using cell lines to more physiologically relevant conditions, particularly the use of transgenic animals, to unveil the protein interaction networks and processes governing NKCC2 protein expression and function in renal health and disease.
